# Mixed Diets Reduce the Oxidative Stress of Common Carp (*Cyprinus carpio*): Based on MicroRNA Sequencing

**DOI:** 10.3389/fphys.2019.00631

**Published:** 2019-05-29

**Authors:** Song Yang, Jie Luo, Yalan Long, Jie Du, GangChun Xu, Liulan Zhao, Zongjun Du, Wei Luo, Yan Wang, Zhi He

**Affiliations:** ^1^ College of Animal Science and Technology, Sichuan Agricultural University, Chengdu, China; ^2^ Key Laboratory of Freshwater Fisheries and Germplasm Resources Utilization, Ministry of Agriculture, Freshwater Fisheries Research Center, Chinese Academy of Fishery Sciences, Wuxi, China

**Keywords:** rice-fish mode, diets, ecological aquaculture, animal welfare, microRNA sequencing, oxidative stress

## Abstract

The rice-fish mode, a mode of ecological aquaculture, has become a popular research topic in recent years. The antioxidant capacity of fish can be affected by the type of diet. Three groups of adult common carp (initial weight 517.8 ± 50 g) were fed earthworm (group A), earthworm + duckweed (group M), and duckweed (group P). The antioxidant capacity of common carp (*Cyprinus carpio*) was evaluated by histopathological sectioning, antioxidant enzyme activity, and the miRNA transcriptome profile. The pathological changes in group M were lighter than those in groups C and A. The activities of superoxide dismutase (SOD) and glutathione peroxidase (GSH-PX) significantly increased in group M, and the malondialdehyde content (MDA) significantly decreased (*p* < 0.05). Additionally, nine differentially expressed miRNAs (DEMs) were found between groups A and M, and eight DEMs found between groups P and M were identified in the liver of common carp. Five miRNAs were reported to be related to oxidative stress, including miR-137-3p, miR-143-3p, miR-146a-5p, miR-21-5p, and miR-125b-5p. Compared with group M, all five detected miRNAs were upregulated in group A, and four of the detected miRNAs were upregulated in group P. The targets of the five miRNAs were further predicted *via* functional analysis. Our study confirmed that omnivorous common carp exhibits stronger antioxidant capacity when feeding on both an animal diet and a plant diet.

## Introduction

The common carp (*Cyprinus carpio*) is a typical omnivorous fish in China (Li et al., 2013); therefore, breeding common carp in paddy fields can make full use of their biological characteristics. Today, food safety and environment-friendly issues of aquaculture are widely considered. Animal welfare not only is related to human health and environmental friendliness but also has a profound impact on the healthy development of livestock and the poultry breeding industry. Compared with developed countries in Europe and America, animal welfare in China still lags behind (Yang et al., 2017b). Ecological aquaculture has important practical significance for ensuring food safety and realizing sustainable development, among which rice field culture is an important mode (Fang et al., 2016). Paddy fields contain both plant and animal diets, and omnivorous fish can choose different types of diets to achieve better animal welfare (Chen and Jiao, 1997). When the environmental factors change, this affects the natural food chain in the paddy field (Boujard et al., 1990). In China, farmers place the adult common carp into paddy fields to improve the quality of fish and to obtain high economic benefits. However, it is not clear whether the ecologically cultured common carp exhibits better antioxidant capacity.

The non-specific immunity of fish plays a more important role in the resistance to the external pathogens, compared with the higher vertebrates (Zapata et al., 2006; Kiron, 2012). Reactive oxygen species (ROS) are products of normal cellular metabolism, which can either be harmful or beneficial to living systems (Valko et al., 2007). The normal metabolism and immune processes of the teleost immune cells produce a large number of ROS (Salton and Ghuysen, 1959). Excessive ROS will cause oxidative stress, resulting in oxidative damage of fish immune organs (Ding et al., 2013). In addition, fish membrane phospholipids contain a large number of polyunsaturated fatty acids, which are very vulnerable to ROS attack (Tanaka et al., 2017).

The fish antioxidant enzymes, including superoxide dismutase (SOD), catalase (CAT), and glutathione peroxidase (GSH-PX), play important roles in protecting immune organs against ROS attacks (Li et al., 2008). MDA is the final product of lipid peroxidation, which can destroy the structure and function of membranes (Sun et al., 1999). The activities of SOD, GSH-Px, and CAT and the content of MDA are the main indexes to measure fish antioxidant capacity (Szczubiał et al., 2004). Diets can affect the immunity of aquatic animals, including immune enzyme activity, antioxidant enzyme activity, immune parameters (serum protein and superoxide anion production), and immunoglobulin M content (Chan et al., 2008; Yildirim-Aksoy et al., 2010; Li et al., 2016b).

MicroRNA (miRNA) is an abundant class of small (normally 18–25 nt) nucleotides and long single-stranded non-coding RNA (He et al., 2015). At the level of translation, miRNA can normally guide the negative regulation of the expression of target mRNA or degrade the target mRNA by combination with the mRNA 3′-untranslated regions (UTR; Forman et al., 2008). In eukaryotic cells, miRNAs can regulate more than 30% of the target genes and partake in multiple regulatory pathways, including development, immune response, cancer, cell differentiation, and others (O’Connell et al., 2012). The expression of miRNA was influenced by different environmental stress factors (Brzuzan et al., 2012; Tang et al., 2013). MiRNA can regulate the oxidative stress response of mouse, humans, and fish (Mateescu et al., 2011; Tang et al., 2013). In some cases, homeostasis can be achieved through miRNA regulation (Leung and Sharp, 2010).

We have already proven that the paddy field farmed loach has a stronger ability to evoke an intestinal immune response (Yang et al., 2017a). However, determining whether diet is one of the influencing factors requires further study. In this study, earthworms and duckweeds, which are easily obtained and can be eaten by carp in the natural water, were used as animal feed and plant feed to elucidate whether the type of diet affects the antioxidant capacity of omnivorous fish species. The objectives of this study were (1) to observe the specific pathological changes of common carp fed with different types of diets, including vacuole degeneration, fatty degeneration, and spotty necrosis; (2) to detect antioxidant enzyme activities of common carp fed with different types of diets; and (3) to gain an insight into the effects of different types of diets on antioxidation-related expression of miRNAs in the liver of common carp.

## Materials and Methods

### Fish and Sampling

The experimental common carp was obtained from the Chengdu commercial farm (Sichuan, China) and kept in round tanks (2 m × 1 m) of the Hanyang proliferation station (Qingshen, Meishan, Sichuan, China). Fish are all from the same batch (body weight 517.8 ± 50 g) and were randomly divided into three groups (*n* = 60 for each group), with two repetitions in each group. Earthworms and duckweeds are easily obtained and were eaten by carp in the natural water. They were also used as animal and plant diets, respectively. The three groups of common carp were group A (animal diet, earthworm-fed), group M (mixed diets, earthworm:duckweed weight ratio of 1:1), and group P (plant diet, duckweed-fed). Common carp is a typical omnivorous fish, which can feed on both animal diet and plant diet under natural conditions. Therefore, group M was used as the control group. Fish were fed three times a day at 9:00, 13:00, and 17:00. The feeding rate for each day is 4% of body weight. To ensure that the fish in group M could simultaneously ingest earthworm + duckweed (weight ratio 1:1), we fed the fish four times at each time point, approximately 10 min apart. At each feeding time, we first used earthworms to feed the fish one-fourth of the total amount of the diet, and then, approximately 10 min later, we used duckweed to feed the fish another one-fourth of the total amount of the diet. We then used earthworms to feed the fish an additional one-fourth of the total feed amount and finally used duckweed to feed the fish the remaining one-fourth of the feed amount. To rule out the effect of different feeding methods on fish, groups A and P also fed fish four times at each time point, approximately 10 min apart. This feeding method was adopted for both the temporary rearing and the formal trial. The trial was operated in a circulating aquarium system with 21 ± 1°C natural photoperiod for 2 weeks of temporary rearing and 8 weeks of formal trial.

After 2 weeks of temporary rearing, all fish fasted for 24 h, and the initial body weights were recorded. The formal trial lasted for 8 weeks, with the fish growth index being measured every 2 weeks to recalculate the feeding amount. Deaths of experimental fish were recorded during the experiment. During the feeding period, the numbers of dead fish were recorded. Fish were anesthetized by 0.02% tricaine methane sulfonate (MS-222) before decapitation. Thirty experimental fish in each tank were rapidly dissected for liver tissue, and the liver tissues were divided into three parts. One part was collected and fixed in fresh Bouin’s solution for histological analysis, and the other two parts were frozen and stored at −80°C for antioxidant enzymatic activity, lipid peroxidation measurement assay, and miRNA-seq analysis. All experimental protocols were approved by the Animal Care Advisory Committee of Sichuan Agricultural University, Sichuan, China, under permit No. DKY-B20121403.

### Hepatic Histopathological Sections

Five fish from the three groups were randomly selected for histopathological section analysis. First, the randomly selected liver samples fixed in the Bouin’s solution were dehydrated by an automatic dehydrator, and then the samples were embedded into paraffin wax. The embedded samples were sliced into 6 μm thickness using a slicer (Leica 2016, Germany), then the sections were dewaxed and stained with hematoxylin and eosin. After labeling, the sections were sealed with neutral gum. Finally, the images were recorded using the microphotography system (BA400Digital, McAudi Industrial Group Co., LTD.) at 400× magnification.

### Hepatic Antioxidant Enzymatic Activity and Lipid Peroxidation

Six fish from the three groups were randomly selected for antioxidant enzymatic activity and lipid peroxidation analysis. Liver samples were homogenized in cold sodium phosphate buffer (0.1 M, pH 7.0, 4°C) at a ratio of 1:9 (m/v) on ice, and then centrifuged (3–18 K, Sigma®, Germany) at 3,000 × *g* for 10 min at 4°C. After centrifugation, the supernatants were stored at −80°C until use. The detection kits were purchased from the Nanjing Jiancheng Bioengineering Institute (Nanjing, China), and the specific detective methods were performed according to instructions of each kit, including SOD, CAT, and GSH-PX and the content of MDA.

The activities of SOD, CAT, and GSH-PX were determined in a 37°C water bath. The principle of SOD activity determination is that riboflavin can be reduced by light in the presence of oxidizing substances, and the reduced riboflavin can be easily oxidized under aerobic conditions to produce superoxide anions, which can reduce nitroblue tetrazolium (NBT) to blue methyl hydrazone, which exhibits maximum absorption at 560 nm. SOD can remove superoxide anion and inhibit the formation of methyl hydrazone. The principle of CAT activity determination is that the reaction of CAT to decompose H_2_O_2_ can be rapidly quenched by adding ammonium molybdate, and the remaining H_2_O_2_ can react with ammonium molybdate to produce a pale-yellow complex. After the reaction, the OD value of the sample at the wavelength of 405 nm is determined by a microplate analyzer. The principle of GSH-PX activity determination is that GSH-Px can catalyze the oxidation of glutathione (GSH) with benzoic acid chromogenic solution to form a yellow anion, and then the concentration of this anion was determined by microplate analyzer at 422 nm. The content of MDA was determined after incubation in a 95°C water bath, and the principle is that the MDA in lipid peroxide degradation products can condense with thiobarbituric acid (TBA) to form red products with a maximum absorption peak of 532 nm.

### Construction and Sequencing of Small RNA Libraries

According to the manufacturer’s protocol, total RNA was isolated from the liver using Trizol Reagent (Invitrogen, Carlsbad, CA, USA), and integrity and concentration were assessed using the RNA Nano 6,000 Assay Kit of the Bioanalyzer 2,100 system (Agilent Technologies, CA, USA). Total RNA was separated by 15% agarose gels to extract the small RNA (18–30 nt). After being precipitated by ethanol and centrifugal enrichment of small RNA samples, the library was prepared according to the method and process of the Small RNA Sample Preparation Kit (Illumina, RS-200-0048): two fish were sequenced in each group for a total of six samples. The qualified libraries were sequenced by an Illumina HiSeq 2,500 platform to generate 50 bp single-end reads.

### Processing and Mapping of miRNA Sequencing Data

Clean reads were screened from raw sequencing reads by removing low quality-reads, repeating sequences and those lacking 3′-adaptors, and 5′-adaptor contaminant bases at both ends of microRNA reads. Sequences shorter than 18 nt or longer than 32 nt, after trimming, were removed. The filtered reads were processed into different read lengths for further analysis. The high-quality clean reads were mapped to the carp genome (*C. carpio* from http://www.carpbase.org/download_home.php) listed in miRBase 21 using bowtie2 (2.2.3; Langmead and Salzberg, 2012; Kozomara and Griffithsjones, 2014).

### Identification of Differentially Expressed miRNAs and qRT-PCR Verification

We used edgeR to identify differentially expressed miRNAs, with criteria of FDR < 0.05 and |log2-fold change| ≥ 1 to determine the differentially expressed miRNAs (Robinson et al., 2010). The counts of the identified miRNAs in all libraries were normalized as reads per million (RPM). The normalization formula used was as follows:

$$\mathrm{RPM}=\left(\mathrm{actual miRNA count}/\mathrm{total count of clean reads}\right)\;\;\times \;\;10^{6}$$

Total RNAs were isolated using Trizol Reagent (Invitrogen, Carlsbad, CA, USA), followed by cDNA generation using 1 μg of total RNA by miRcute Plus miRNA First-Strand cDNA Synthesis kit (Tiangen, Beijing, China). Quantitative real-time PCR (qRT-PCR) was performed on a CFX Connect Real-Time system (Bio-Rad Laboratories, USA) using the qRT-PCR Detection Kit (TANGEN). The primers and annealing temperature are shown in additional file 4. PCR amplification was conducted *via* an initial denaturation at 95°C for 15 min, 40 cycles of amplification including the denaturation at 94°C for 20 s, annealing for 30 s, and extension at 72°C for 30 s. Each sample was tested in triplicate. Finally, the melting curve was acquired to verify the specificity of PCR amplification. U6 was used as an internal control. The levels of 11 miRNAs were calculated using the 2^−ΔΔCT^ method (Livak and Schmittgen, 2001).

### Target Analysis of the miRNAs Related to Oxidative Stress

The targets of differentially expressed miRNA were predicted using mirPath v.3 (Vlachos et al., 2015). The Gene Ontology (GO, http://www.geneontology.org/) and Kyoto Encyclopedia of Genes and Genomes (KEGG, http://www.genome.jp/kegg/) databases were used for functional analysis of the predicted target genes. Significantly enriched GO terms and KEGG pathways in DEGs as compared to the genome background were defined by hypergeometric testing. When GO terms and KEGG pathways exhibited *p* < 0.05, they were defined as significantly enriched GO terms and KEGG pathways in DEGs.

### Statistical Analysis

SPSS20.0 software (SPSS, Chicago, IL, USA) was used for analysis, to analyze the results of survival rate, antioxidant enzyme activity, MDA content, and qRT-PCR (SPSS, Chicago, IL, USA). Significant differences were found using one-way analysis of variance (ANOVA), followed by Fisher’s least significant difference *post hoc* test and Duncan’s multiple range tests, after confirming data normality and homogeneity of variances. Differences were considered to be significant if *p* < 0.05.

## Results

### Hepatic Histopathological Analysis

**Figure 1:**
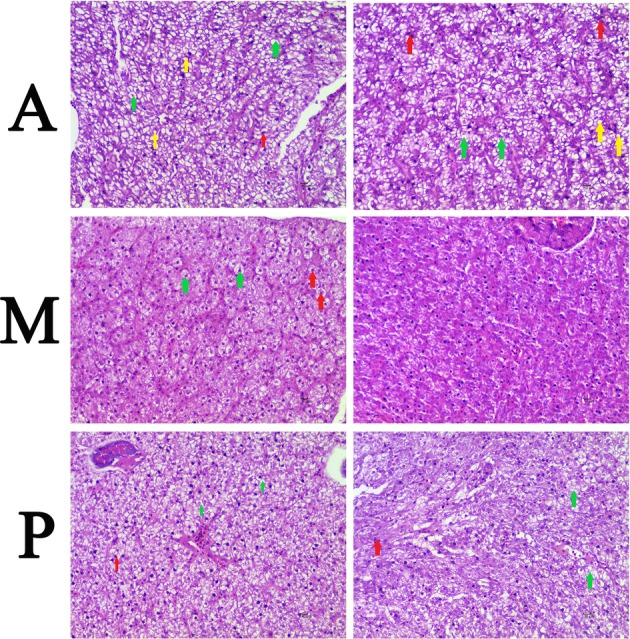
Hepatic histopathological changes of common carp fed with different types of diets (×400). (A) Common carp fed with earthworm. (M) Common carp fed with earthworm and duckweed. (P) Common carp fed with duckweed. Note: green arrow showing hepatocyte vacuole degeneration; yellow arrow showing fatty degeneration; and red arrow showing spotty necrosis.

All fish survived the experiment. The hepatic histological observation (×400) was shown in Figure 1. Five experimental fish were randomly selected from each group for histopathological observation. In group A, fatty degeneration was found in parts of liver cells of two experimental fish; vacuole degeneration was found in five experimental fish; and spotty necrosis was found in multiple hepatocytes of two experimental fish. In group M, vacuoles degeneration was found in one experimental fish, and spotty necrosis was found in a small number of hepatocytes in one experimental fish, with no obvious fatty degeneration. In group P, vacuolar degeneration was found in four experimental fish, and spotty necrosis was found in a small number of hepatocytes in two experimental fish, with no obvious fatty degeneration. In summary, only some of the hepatocytes of the common carp fed earthworms displayed fatty degeneration, and the pathological changes in group M were relatively mild.

### Hepatic Antioxidant Enzyme Activities and Lipid Peroxidation Analysis

**Figure 2:**
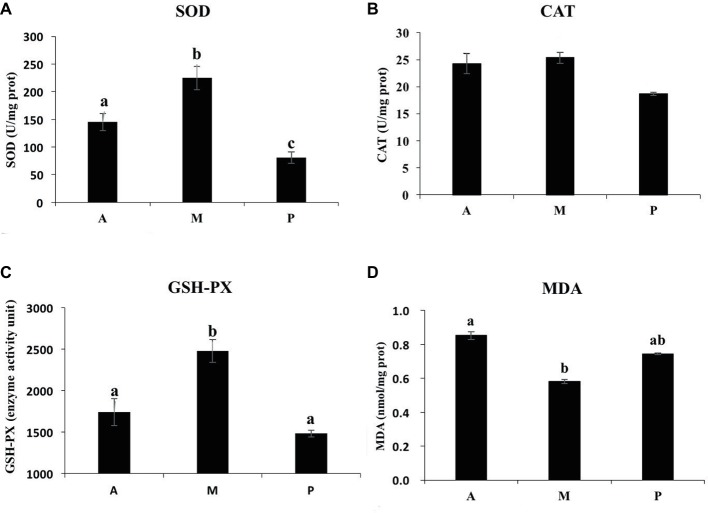
Effects of different baits on antioxidant responses in liver of common carp. **(A)** SOD activity; **(B)** CAT activity; **(C)** GSH-PX activity; and **(D)** contents of MDA. ^a,b,c^Not sharing a common superscript letter was significantly different (*p* < 0.05) as determined by Duncan’s multiple range test. Values are means ± SD, *n* = 6.

As shown in Figure 2, the common carp fed with earthworm + duckweed (group M) exhibited significantly enhanced SOD and GSH-PX activity (*p* < 0.05), but no significant effect on CAT activity was observed (*p* > 0.05). The SOD activity in group A was significantly higher than in group P (*p* < 0.05). GSH-PX and CAT activities had no significant differences between groups A and P (*p* > 0.05). The content of MDA in group M was significantly lower than in group A (*p* < 0.05) but had no significant difference versus group P (*p* > 0.05).

### Hepatic miRNA Libraries

We built microRNA libraries by using total RNA and filtered 20,868,000; 18,984,242; 22,300,487; 19,538,683; 17,110,676; and 14,747,090 high-quality reads, which were obtained from samples A1, A2, M1, M2, P1, and P2, respectively, of which 14,630,781 (70.1%); 11,078,781 (58.4%); 15,194,709 (68.1%); 14,065,615 (72%); 12,057,299 (70.5%); and 11,343,210 (76.9%) were mapped to the reference genome (Table 1). The majority size of the microRNAs was in the range from 18 to 36, with 22 nt as the most frequent size (Supplementary Figure S1).

**Table 1:** Summary statistics of the miRNA sequencing results for the six common carp sample libraries.

Sample	A1	A2	M1	M2	P1	P2
Clean reads	20,868,000	18,984,242	22,300,487	19,538,683	17,110,676	14,747,090
Mapped reads	14,630,781	11,078,781	15,194,709	14,065,615	12,057,299	11,343,210
Mapping rate (%)	70.1	58.4	68.1	72	70.5	76.9
Known miRNAs	225	225	222	227	228	221

### Differential Expression Analysis of Known MicroRNAs

In total, we discovered 236 known carp miRNAs (225, 225, 222, 227, 228, and 219 in A1, A2, M1, M2, P1, and P2, respectively), and 201 (85.17%) of the known miRNAs were detected in six libraries (Table 1 and Supplementary Table S1).

**Figure 3:**
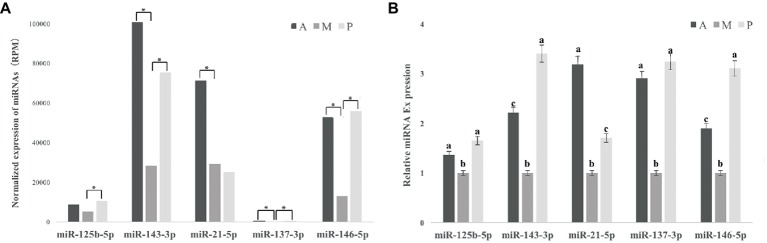
Effects of different baits on expression level of miRNAs related to oxidative stress in liver of common carp *via* miRNA-seq and qRT-PCR. **(A)** Five antioxidant-related miRNAs validated using miRNA-seq. * is considered to indicate differential expression of miRNA between groups A and M or groups P and M. **(B)** Five antioxidant-related miRNAs validated using qRT-PCR. ^a,b,c^Not sharing a common superscript letter was significantly different (*p* < 0.05) as determined by Duncan’s multiple range test. Values are means ± SD, *n* = 6. MiR-137-3p, miR-143-3p, miR-146a-5p, miR-21-5p, and miR-125b-5p were reported to be related to oxidative stress, and the expression level of five known microRNAs *via* miRNA-seq had highly similar trend with the relative expression level of five known microRNAs *via* qRT-PCR.

For the known miRNA, nine differentially expressed miRNAs (DEM) were found between groups A and M, including miR-137-3p, miR-143-3p, miR-146a-5p, miR-20a-3p, miR-21-5p, miR-218a-3p, miR-457b-3p, miR-142-3p, and miR-192-5p: among them, only miR-192-5p was downregulated in group A (Table 2). We identified eight known miRNAs differentially expressed between P and M, including miR-125b-5p, miR-137-3p, miR-143-3p, miR-146a-5p, miR-217-5p, miR-365-3p, miR-551-3p, and miR-7,133-3p, and only miR-217-5p was downregulated in group P (Table 2). MiR-137-3p, miR-143-3p, miR-146a-5p, miR-20a-3p, miR-21-5p, miR-457b-3p, miR-125b-5p, miR-217-5p, miR-365-3p, miR-551-3p, and miR-7,133-3p were selected for validation *via* qRT-PCR analysis, and the expression levels of 11 known microRNAs *via* qRT-PCR showed highly similar trends with the relative expression level of 11 known microRNAs *via* miR-seq (Figure 3, Supplementary Table S2, Supplementary Figure S2).

**Table 2:** Differentially expressed miRNAs (DEMs) between groups A and M and groups P and M.

miRNA	Mean reads per million (M)	Mean reads per million (A or P)	Log2-fold change (A or P/M)	Regulation	*p*	FDR
**DEMs between A and M**						
miR-137-3p	0.001	9.91	13.27	Upregulated	0.00	0.02
miR-143-3p	28264.64	100821.41	1.83	Upregulated	0.00	0.00
miR-146a-5p	12962.54	52756.40	2.02	Upregulated	0.00	0.00
miR-20a-3p	59.84	355.41	2.57	Upregulated	0.00	0.04
miR-21-5p	29013.31	71255.68	1.30	Upregulated	0.00	0.00
miR-218a-3p	0.06	23.04	8.71	Upregulated	0.00	0.02
miR-457b-3p	0.49	19.86	5.35	Upregulated	0.00	0.00
miR-142-3p	377.38	1084.43	1.52	Upregulated	0.00	0.04
miR-192-5p	401604.03	195106.12	−1.04	Downregulated	0.00	0.00
**DEMs between P and M**						
miR-137-3p	0.001	18.61	14.18	Upregulated	0.00	0.03
miR-143-3p	28264.64	75279.35	1.41	Upregulated	0.00	0.00
miR-146a-5p	12962.54	55778.44	2.11	Upregulated	0.00	0.00
miR-125b-5p	5173.78	10444.81	1.01	Upregulated	0.00	0.03
miR-217-5p	6725.2	2519.51	−1.41	Downregulated	0.00	0.00
miR-365-3p	0.001	38.54	15.23	Upregulated	0.00	0.02
miR-551-3p	25.82	146.69	2.51	Upregulated	0.00	0.00
miR-7,133-3p	87.24	340.73	1.97	Upregulated	0.00	0.03

### Effects of Different Diets on miRNA Expression of Common Carp Liver Related to Oxidative Stress

MiR-137-3p, miR-143-3p, miR-146a-5p, miR-21-5p, and miR-125b-5p were reported to be related to oxidative stress, and the expression levels of five known microRNAs *via* miRNA-seq showed highly similar trends with the relative expression levels of five known microRNAs *via* qRT-PCR (Figure 3). The relative expression of five miRNAs of common carp fed with earthworm + duckweed was significantly downregulated *via* qRT-PCR (*p* < 0.05).

### Prediction and Bioinformatics Analysis of Target Genes

**Figure 4:**
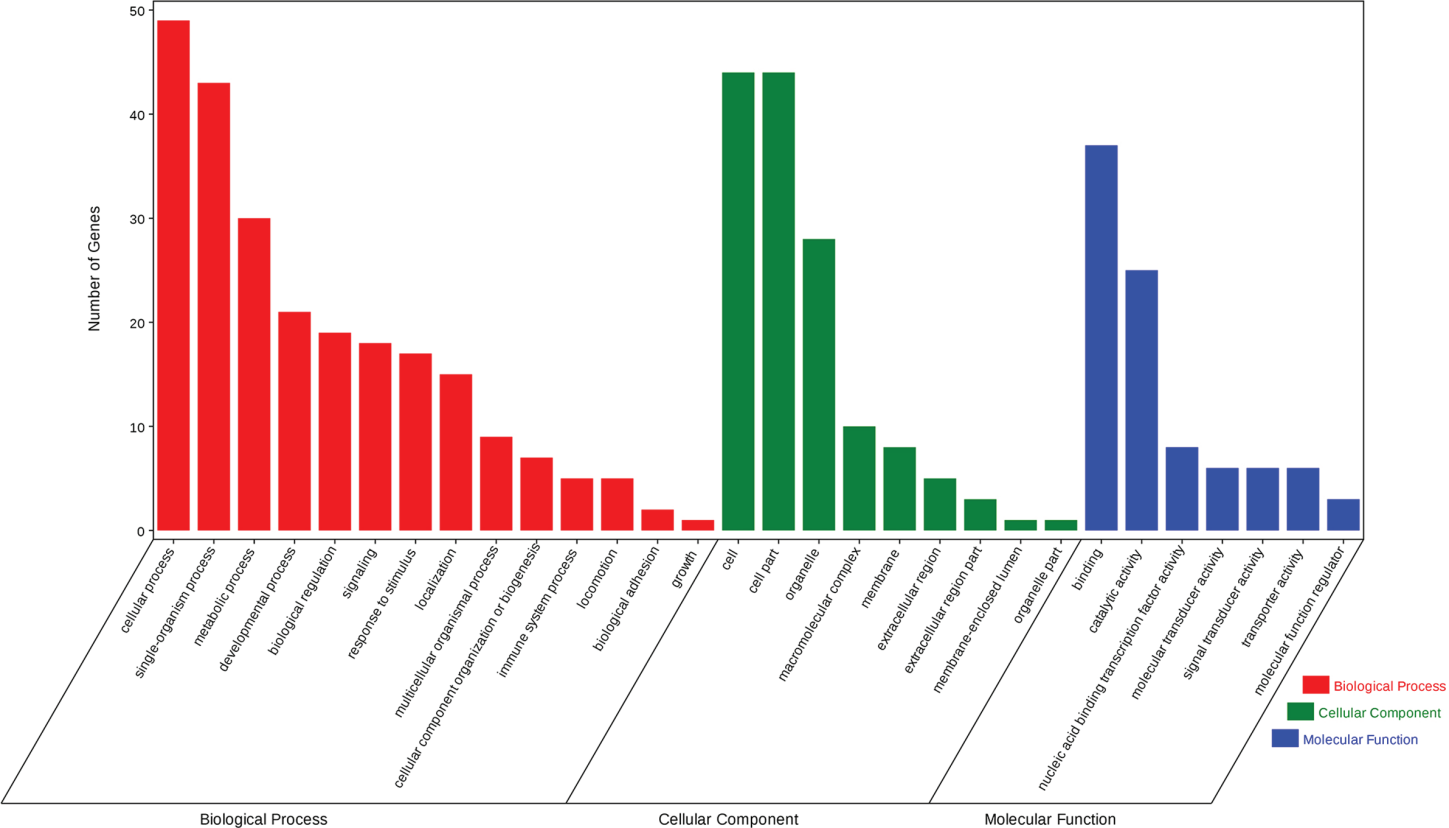
Differentially expressed miRNAs related to oxidative stress target gene-related gene ontology (GO) terms. GO enrichment analysis showed that 127 targets genes attended 14 GO terms of biological process (BP), including immune system process, response to stimulus, metabolic process, and so on; seven GO terms of the molecular function (MF), including catalytic activity, transporter activity, molecular transducer activity, and so on; nine GO terms of cellular component (CC), containing membrane-enclosed lumen and macromolecular complex.

**Figure 5:**
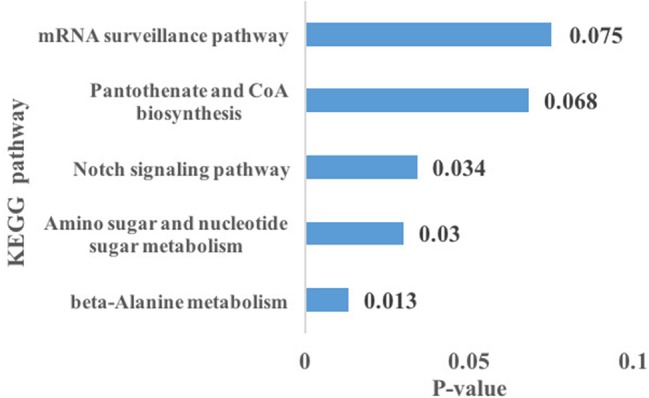
Differentially expressed miRNAs related to oxidative stress target gene-related top five Kyoto Encyclopedia of Genes and Genomes (KEGG) pathways, among that, β-alanine metabolism, amino sugar, and nucleotide sugar metabolism, and the Notch signaling pathway were significantly enriched (*p* < 0.05 terms).

Using the prediction program, the five DEMs related to oxidative stress were connected with 127 target genes. GO enrichment analysis showed that target genes corresponded with 14 GO terms of biological processes (BP), including immune system processes, response to stimulus, metabolic processes, and so on; seven GO terms of the molecular function (MF), including catalytic activity, transporter activity, molecular transducer activity, and others; nine GO terms of cellular components (CC), containing membrane-enclosed lumen and macromolecular complex (Figure 4). KEGG pathway analysis showed that three significantly enriched pathways (*p* < 0.05 terms), β-alanine metabolism, amino sugar, and nucleotide sugar metabolism pathways, are related to metabolism, and the Notch signaling pathway is related to environmental information processing. In addition, the results also include pantothenate and CoA biosynthesis, mRNA surveillance pathway, pyrimidine metabolism, and so on. The top five KEGG enrichment terms were shown in Figure 5.

## Discussion

### Hepatic Histopathological Changes

Liver is a central immune organ, the center of energy metabolism of fish, and also an important excretory organ (Camargo and Martinez, 2007; Heymann and Tacke, 2016). The histopathological changes of liver will be affected by environmental stress factors, such as dietary lipid levels (Qi et al., 2012), vitamin C deficiency (Chi et al., 2008), dietary cadmium (Luo et al., 2016), hypoxia (Cannito et al., 2014), and so on. Fatty degeneration is the accumulation of triglycerides in the cytoplasm. Diet is a common cause of fatty degeneration of the liver (Weber et al., 2008). The degree of fatty degeneration in the liver cells of redfish (Sciaenops ocellatus) was found to be proportional to the dietary fat level (Feng and Jia, 2005). Fatty degeneration was found only in the hepatocytes of common carp fed earthworms, probably because earthworms had higher fat content than duckweeds. The integrity of liver tissue structure is an important indicator of liver health and a prerequisite to ensure its normal function. When certain pathological changes occur in fish liver tissue, the health of fish will be affected, and when the pathological changes reach a certain level, the mortality of fish will increase (Wang et al., 2013). In this study, the pathological changes of livers of common carp fed with earthworms and duckweeds were relatively mild, and the fish were healthier.

### Hepatic Antioxidant Enzymes and Lipid Peroxidation

Antioxidant enzymes can be used as indicators of antioxidant status and also can even be used as biomarkers of oxidative stress (Li et al., 2011), including GSH-Px, CAT, and SOD (Zhao et al., 2013). In the present study, SOD and GSH-Px activities in the livers of common carp fed only with duckweed or earthworm were significantly decreased. The content of MDA directly reflects the state of lipid peroxidation, and the higher the content of MDA, the greater the oxidative damage to the body (Jiang et al., 2011). The content of MDA in the earthworms + duckweed group was the lowest, but there was no significant difference between the earthworm + duckweed group and the duckweed group. Changes in the activities of SOD and GSH-Px and the contents of MDA indicated that the common carp fed with mixed diets might have higher antioxidant capacity and suffer lower oxidative damage. Common carp is a typical omnivorous fish in its natural state. We speculate that mixed diets can better meet the nutritional needs of common carp and enable the fish to attain a healthy physiological state. The mandarin fish (Siniperca chuatsi) hybrid fed live diets have been reported to exhibit higher antioxidant enzyme activities and lower MDA contents than the fish fed formulated diets (Li et al., 2017). Snakehead (Channa argus) was fed with frozen fish and formulated diet. It was found that the frozen fish group had higher SOD activity (Yuan et al., 2017). Both mandarin fish and snakehead are typical carnivorous fish, and live food is more nutritious and beneficial to the digestion and absorption of carnivorous fish. SOD, GSH-Px, and CAT are the main protective enzyme systems in vertebrates. Under normal circumstances, the three enzymes combine to clear reactive oxygen species and protect animals from free radicals (Sun et al., 1999). SOD can catalyze the superoxide anion (O_2_^−^) disproportionation reaction to generate H_2_O_2_ and O_2_ (Jianxiao et al., 2011), and the H_2_O_2_ is mainly removed from the body by CAT and GSH-Px. Catalase activity was the highest in group M, but there was no significant difference among the three groups. When common carp were exposed to different types of diets, the CAT activity of liver was inhibited at 56 days. We speculated that SOD catalyzed the superoxide anion (O_2_^−^) disproportionation reaction to generate H_2_O_2_, which was mainly removed by GSH-Px at this time. When swamp eel (*Monopterus albus*) was stressed by Cu2^+^, it was found that the CAT activity of liver was inhibited at 96 h, and the activity of GSH-Px was significantly increased. At this time, GSH-Px played a major antioxidant role (Lu et al., 2002). In 2013, Tang et al. found that vitamin deficiency caused a significant decrease in SOD activity in the liver of Nile tilapia (*Oreochromis niloticus*) compared with the control group, but there was no significant difference in CAT enzyme activity, possibly because the CAT enzyme was less sensitive to VE deficiency (Tang et al., 2013).

### Hepatic Antioxidant-Related miRNAs

In this study, all average sizes of sequences of groups A, M, and P were 22 nt, which is consistent with typical miRNA sizes in the grass carp (*Ctenopharyngodon idellus*; Xu et al., 2015), Nile tilapia (*O. niloticus*; Yan et al., 2014), *Atlantic salmon* (*Salmo salar*; Barozai, 2012), and so on. Among DEMs, miR-137-3p, miR-143-3p, miR-146a-5p, miR-21-5p, and miR-125b-5p were reported to be related to oxidative stress. miRNA is involved in the regulation of oxidative stress mainly by binding to specific junctions and sites of mRNA, eventually leading to blocked translation or direct degradation of target genes (Huang et al., 2011).

The small regulatory RNA miR-137 plays a crucial role in biological functions and diseases, including cell proliferation, differentiation, cancer, and cardiovascular diseases (Zhang et al., 2010; Shin et al., 2014; Wang et al., 2015). It was found that miR-137 was upregulated during the process of H_2_O_2_-induced apoptosis, and miR-137 exerts a specific function mainly through cell division control protein 42 homolog (CDC42; Wang et al., 2015). In 2016, Li et al. further found that downregulation of miR-137 ameliorates HG-induced injury in HUVECs by overexpression of AMPKα1, leading to increased cellular reductive reactions and decreased oxidative stress (Li et al., 2016a). These results indicate that miR-137 was linked to oxidative stress, and downregulation of miR-137 can reduce oxidative stress. In the present study, the expression of miR-137-3p was observed to be downregulated in the liver of common carp fed with duckweed + earthworm, suggesting that mixed diets could reduce oxidative stress of common carp. We speculate that because common carp is a kind of omnivorous carp in its natural state, mixed bait can better meet its nutritional needs for animal and plant bait, enabling the fish to achieve a healthier state.

MiR-143 is involved in the regulation of several cellular processes including proliferation, migration, and chemoresistance (Chen et al., 2009; Qian et al., 2013). A previous study found that overexpression of miR-143 can aggravate apoptosis and inhibition of proliferation (Borralho et al., 2011). In 2017, Xu et al. treated the human normal liver cell line L02 with H_2_O_2_, finding that miR-143 expression increased after oxidative stress injury, and it was further discovered that downregulation of miR-143 protects L02 cells from apoptosis by adjusting the expression levels of HK2 and ADRB1 (Xu et al., 2017). In 2018, Gomes et al. found that overexpressed miR-143 can increase oxidative stress by reducing antioxidant enzyme superoxide dismutase 1 (SOD1) expression and increasing reactive oxygen species (ROS) generation (Gomes et al., 2018). In the present study, the expression of miR-143 was upregulated, and the activity of SOD was decreased when the carp was fed a single type of diet, indicating that the fish in the single diet group may have suffered oxidative stress injury. Interestingly, the inverse tendencies of miR-43 expression and SOD activity were observed in the mixed diet group, possibly because the mixed diet was more suitable for omnivorous fish.

Target gene analysis showed that miR-146a was involved in the response to oxidative stress (Zheng et al., 2014). Changes in the expression of miR-146a have been implicated in disease mechanisms that may be related to oxidative stress (Babar et al., 2008). Some studies reported that the increased expression of miR-146a was significantly related to environmental metal contents, such as cadmium and aluminum sulfate (Pogue et al., 2009; Bollati et al., 2010). In 2013, Tang et al. found that upregulation of miR-146a was found in the liver of tilapia with excessive supplementation of VE and concluded that miR-146a expression levels are associated with oxidative stress. In the current study, compared with the mixed diet group, miR-146a was significantly upregulated in the liver of common carp fed a single type of diet. It may, therefore, be concluded that the fish in the single diet group may have suffered oxidative stress injury.

MiR-21 was found to be aberrantly expressed in multiple cancers (Krichevsky and Gabriely, 2010). It also plays a vital role in cancer, cardiovascular disease, and inflammation, mainly by targeting many tumor suppressor genes related to proliferation, apoptosis, and invasion (Pan et al., 2010). Oxidative stress induced by ionizing radiation and excessive supplementation of VE can increase the expression of miR-21 (Yoke-Kqueen, 2011; Tang et al., 2013). The expression of miR-21 was negatively correlated with T-AOC, SOD, and CAT (Tu et al., 2014). These results are in agreement with those of the present study, suggesting that the common carp fed with earthworms may have suffered oxidative stress.

MiR-125b expression is increased by an oxidative stress-dependent mechanism, which is similar to that of miR-21 (Manca et al., 2011). Previous studies proved that miR-125b was upregulated by excessive supplementation of VE (Tang et al., 2013). In our study, the expression level of miR-125b in common carp fed with duckweed + earthworm was lower than earthworm or duckweed groups, but it was only significantly lower than that of common carp fed with duckweed. This indicates that omnivorous carp may maintain better health status when fed both plant and animal feed.

### Ecological Aquaculture and Animal Welfare

Much attention has been paid to ecological aquaculture and animal welfare, and the former has become a hot research topic in recent years. Ecological aquaculture is a cultivation industry mode that emphasizes the integrity, coordination, and participation of ecological ecosystems. It promotes the sustainable development of aquaculture (Andersen et al., 2015), such as rice field culture. Fisheries in large waters are being gradually transformed into ecological fisheries with the aims of environmental protection and natural diet utilization. Carp is the main fish of paddy fields in China (Andersen et al., 2015). In domestic production, farmers like to add adult common carp into the rice fields to promote better environmentally adaptive capacity. To some extent, the survival rate in our study can partly reflect this situation. At present, more than 100 countries around the world have established laws and regulations regarding animal welfare. Animal welfare refers to the benign state in which the difference between animal needs and living environment is minimized (Broom, 1996). Animal welfare primarily includes two aspects of improving the culture environment and stress management, and whether animals produce a stress response is an indicator of its effectiveness (Yang et al., 2017b). In the present study, higher antioxidant enzyme activity and lower MDA content and antioxidation-related miRNA expression levels were found for omnivorous carp fed with duckweeds + earthworms versus those fed with single diets, suggesting that rice-field culture is a new and promising farming model, which can provide both animal and plant feed for omnivorous fish. However, the type of diet will be affected by environmental factors (Boujard et al., 1990). In some special cases, it is necessary not only to provide an additional diet to satisfy the requirements of healthy animal growth but also to promote good animal welfare in rice-fish mode. Low density in paddy fields might be considered a probable reason to reduce stress; however, much further study is necessary for verification.

## Conclusion

Common carp farmed in paddy fields may have better antioxidant capacity and achieve better animal welfare due to combined plant diet and animal diet. In the present study, we identified five differentially expressed miRNAs, which are reported to be related to oxidative stress, including miR-137-3p, miR-143-3p, miR-146a-5p, miR-21-5p, and miR-125b-5p. All five detected miRNAs were upregulated by earthworm diet, and four of the detected miRNAs were upregulated by duckweed diet, except miR-21-5p *via* miRNA-seq, and the relative expression levels of five miRNAs of common carp fed with earthworm + duckweed were significantly downregulated *via* qRT-PCR (*p* < 0.05). For omnivorous fish, feed of a single diet may cause inordinate oxidative stress.

## Ethics Statement

All experimental protocols were approved by the Animal Care Advisory Committee of Sichuan Agricultural University, Sichuan, China, under permit No. DKY-B20121403.

## Author Contributions

LZ managed the grants, supervised the laboratory work, and led the design and coordination of this study. SY, JL, and YL conceived and designed the research, performed the study and also drafted the manuscript. ZD and GX participated in the tissue sampling. JD and YW performed the majority of the culture experiments. WL and ZH provided helpful guidance for the statistical analysis. All authors read and approved the final manuscript.

### Conflict of Interest Statement

The authors declare that the research was conducted in the absence of any commercial or financial relationships that could be construed as a potential conflict of interest.
