# Supplementary material for: Mixed Diets Reduce the Oxidative Stress of Common Carp (Cyprinus carpio): Based on MicroRNA Sequencing
Source: Front Physiol. 2019 May 29;10:631. doi: 10.3389/fphys.2019.00631 (PMC6549001; doi:10.3389/fphys.2019.00631)
Supplement: Supplementary file 1 [file Data_Sheet_1.doc]

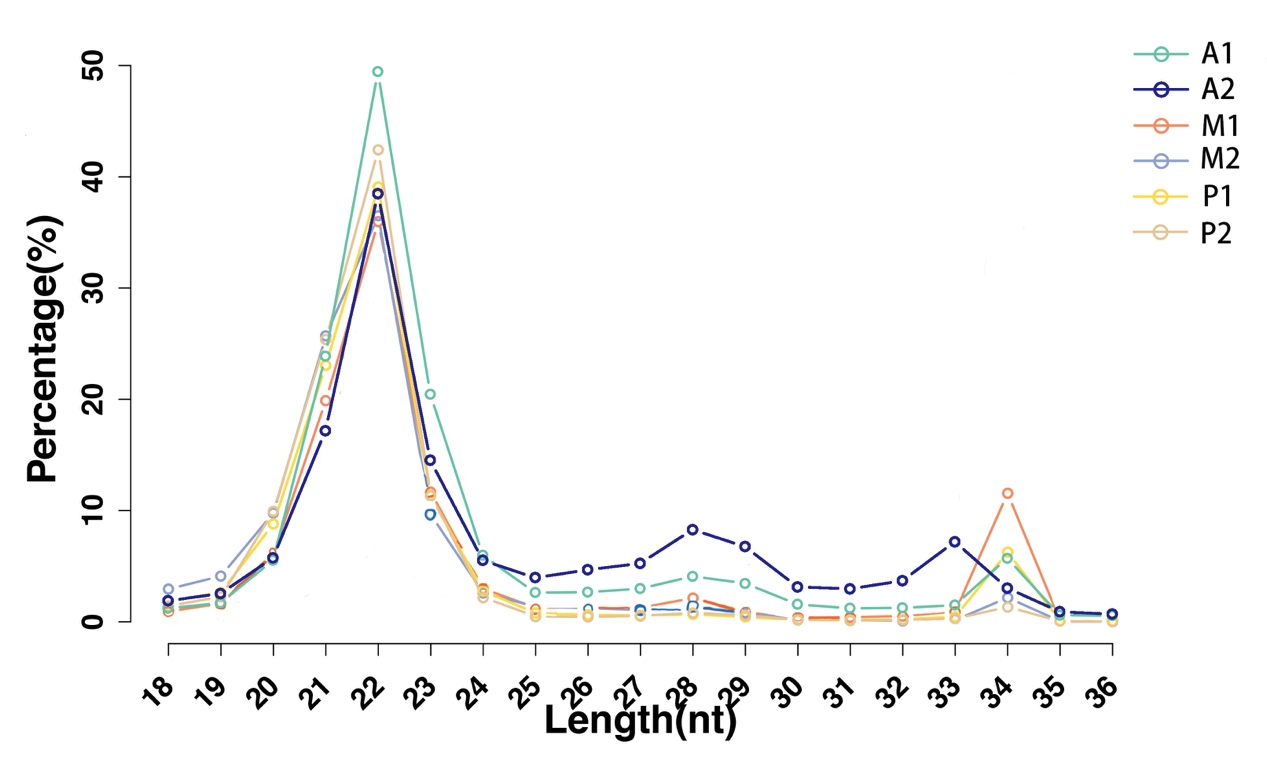


**Figure S1** Length of clean reads of six library. The majority size of microRNAs was in the range from 18 to 36, with 22 nt as the most frequent size.


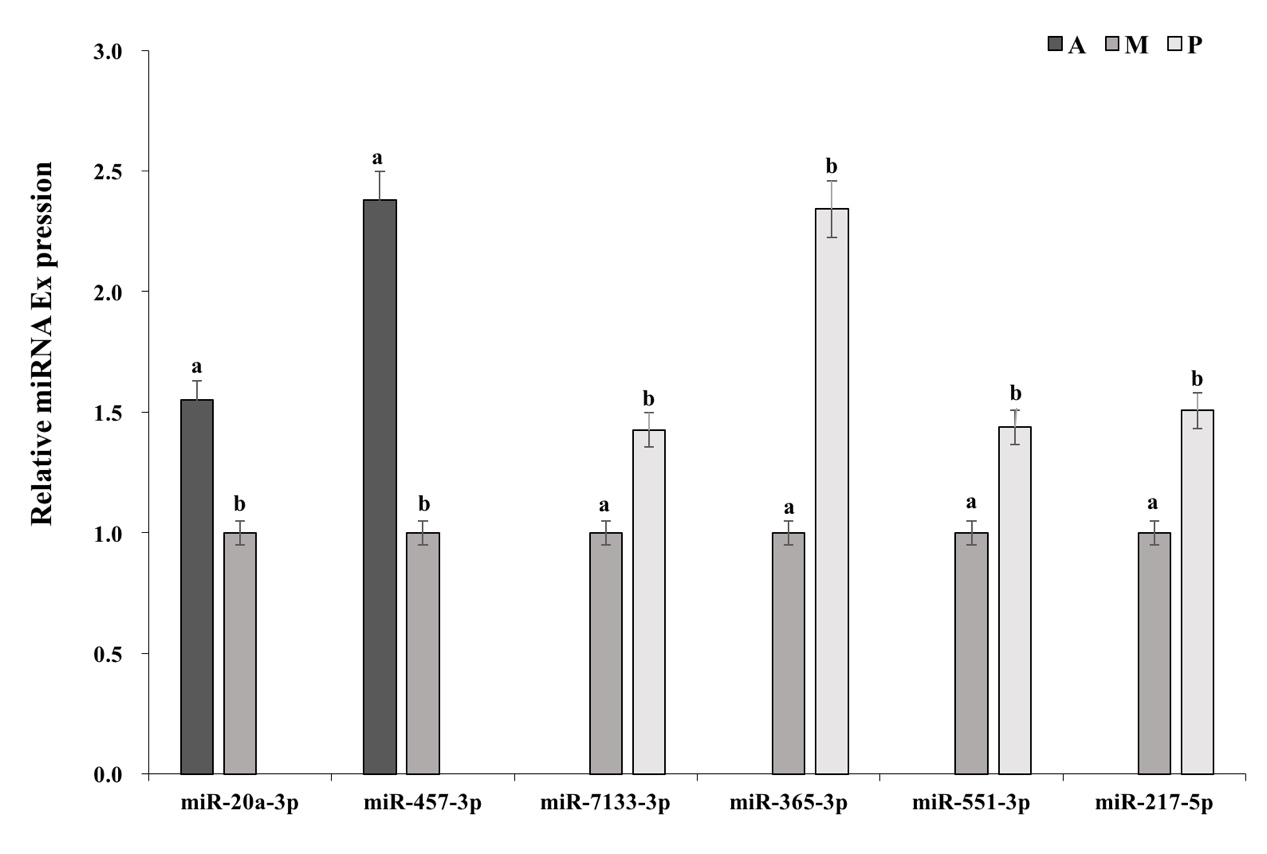


**Figure S2** qRT-PCR relative miRNA expression analysis of miR-457b-3p, miR- 217-5p, miR-365-3p, miR-551-3p, miR-20a-3p and miR-7133-3p. ^a,b,c^ Not sharing a common superscript letter were significantly different (P < 0.05) as determined by Duncan's multiple range test. Values are means ± SD, n =6. The expression level of 6 different expression microRNAs via qRT-PCR had highly similar with miRNA-seq result, except miR-217-5p.

**Table S1** Summary information of 226 known miRNAs in six libraries, and the miRNA expression was normalized to reads per million (RPM) values

| **miRNA** | **Mean Reads per million（A）** | **Mean Reads per million（M）** | **Mean Reads per million（P）** |
| --- | --- | --- | --- |
| miR-20a-5p | 834.2752109 | 623.7034774 | 849.3944297 |
| miR-20a-3p | 355.4045497 | 59.83580011 | 62.43333218 |
| miR-338-5p | 140.4434687 | 138.2413465 | 213.6011409 |
| miR-338-3p | 1758.842217 | 1928.249673 | 2661.151285 |
| miR-205-5p | 399.5512045 | 146.7544227 | 276.2112422 |
| miR-206-3p | 0.293080143 | 0.28341535 | 0.24200632 |
| miR-135c-5p | 8.955411661 | 33.60381862 | 1.331034757 |
| miR-135c-3p | 0 | 0.422814343 | 6.026624995 |
| miR-29b-5p | 52.5059365 | 18.08317403 | 12.97667023 |
| miR-29b-3p | 96.50517282 | 137.5246073 | 226.4062019 |
| miR-29a-5p | 152.9742123 | 118.2598278 | 109.4611818 |
| miR-29a-3p | 1676.808152 | 1520.465138 | 2382.649717 |
| miR-181a-5p | 51631.47667 | 33839.5087 | 24344.90742 |
| miR-181a-3p | 529.705118 | 687.849174 | 356.3703055 |
| miR-181b-5p | 3002.600209 | 1749.562208 | 737.336131 |
| miR-181b-3p | 8.15814425 | 7.792300373 | 6.545313475 |
| miR-181c-5p | 98.15983235 | 45.13219479 | 33.38854471 |
| miR-181c-3p | 0.664545955 | 0.551505592 | 1.178895797 |
| miR-92a-5p | 10.12565356 | 8.860043976 | 5.486240824 |
| miR-92a-3p | 17085.5481 | 19015.68247 | 23831.5065 |
| miR-92b-5p | 1.166654549 | 1.341237294 | 0.400601714 |
| miR-92b-3p | 219.3307309 | 257.4237402 | 315.69529 |
| miR-363-5p | 3.167344223 | 1.782421937 | 3.574279627 |
| miR-363-3p | 768.7550963 | 693.5603896 | 1160.358543 |
| miR-365-3p | 0 | 0 | 38.53950638 |
| miR-459-5p | 9.275313252 | 25.6956027 | 14.61899571 |
| miR-459-3p | 0.567083538 | 1.50362395 | 0.528651315 |
| miR-455-5p | 506.7140227 | 394.0390459 | 584.9771528 |
| miR-455-3p | 63.72552496 | 54.27034982 | 77.14222889 |
| miR-125b-5p | 8667.725979 | 5173.776116 | 10444.81107 |
| miR-125b-3p | 110.6923304 | 49.17417781 | 60.52133734 |
| miR-125c-5p | 609.1480089 | 371.5482533 | 901.775986 |
| miR-125c-3p | 1.173013465 | 0.604357384 | 0.400601714 |
| miR-142-5p | 17688.80897 | 16042.93362 | 26008.64137 |
| miR-142-3p | 1084.422986 | 377.3794213 | 552.5369276 |
| miR-140-5p | 159.5166663 | 117.2301986 | 117.2159032 |
| miR-140-3p | 2542.234278 | 2849.612798 | 2965.179601 |
| miR-148-5p | 2633.209652 | 1912.412767 | 1031.221049 |
| miR-148-3p | 29932.81636 | 22269.69361 | 17963.62586 |
| miR-24-5p | 84.31328464 | 76.21877951 | 139.2596176 |
| miR-24-3p | 256.0087526 | 292.0147322 | 413.1883691 |
| miR-25-5p | 3.322729777 | 3.335066402 | 3.55900673 |
| miR-25-3p | 1403.976588 | 1438.967369 | 1514.865708 |
| miR-734-3p | 0.117232057 | 1.492130119 | 0.24200632 |
| miR-730-5p | 56.62988972 | 114.198398 | 181.1895725 |
| miR-730-3p | 8.638404529 | 12.35985529 | 19.21478939 |
| miR-203b-5p | 1.629223862 | 0.328604589 | 0.400601714 |
| miR-203b-3p | 75.54468444 | 45.17699456 | 29.5129372 |
| miR-153c-5p | 6.424406163 | 4.320880168 | 3.272361734 |
| miR-153c-3p | 507.1293081 | 508.8767714 | 349.2169589 |
| miR-153b-5p | 1.361579383 | 0.959780913 | 0.634381578 |
| miR-153b-3p | 78.36114827 | 123.7834386 | 77.52867044 |
| miR-551-5p | 0.775419097 | 1.011846619 | 0.687836717 |
| miR-551-3p | 63.48714929 | 25.82105044 | 146.686039 |
| miR-726-5p | 9.482263027 | 9.411549568 | 12.20778262 |
| miR-726-3p | 13.25553718 | 14.79948641 | 8.155946787 |
| miR-725-5p | 1.08826888 | 0.22673228 | 5.293593236 |
| miR-725-3p | 31.85923658 | 17.72490363 | 5.233648022 |
| miR-190-5p | 29.09584564 | 20.94483341 | 26.81858551 |
| miR-190-3p | 9.43567194 | 11.23611555 | 6.286854245 |
| miR-192-5p | 195106.1173 | 401604.0304 | 260101.1853 |
| miR-192-3p | 1431.5185 | 1287.185893 | 1727.369594 |
| miR-194-5p | 13017.62861 | 8311.480991 | 10705.33047 |
| miR-194-3p | 13.91800446 | 10.82174985 | 12.03860074 |
| miR-199-5p | 22331.18922 | 22632.63851 | 14010.59158 |
| miR-199-3p | 6279.427904 | 6214.768649 | 9665.859918 |
| miR-10d-5p | 240.1590339 | 541.2669604 | 608.4080742 |
| miR-10d-3p | 0.234464115 | 0.762912763 | 1.170669342 |
| miR-10b-5p | 15085.2939 | 18379.13145 | 12782.28843 |
| miR-10b-3p | 11.47451511 | 13.19015887 | 18.62209646 |
| miR-10c-5p | 10802.30121 | 8422.33656 | 8333.94193 |
| miR-10c-3p | 8.432147645 | 9.299755601 | 8.961836629 |
| miR-214-5p | 45.51664836 | 49.1782074 | 85.96900312 |
| miR-214-3p | 351.5704029 | 254.7624757 | 365.2581377 |
| miR-429-5p | 0.371465812 | 0.05668307 | 0.113366711 |
| miR-429-3p | 406.7020358 | 345.5979167 | 501.57956 |
| miR-196a-5p | 8.221733412 | 3.955534982 | 1.617089746 |
| miR-196a-3p | 0.957626099 | 0.672534286 | 0.279598554 |
| miR-196b-5p | 5.089648228 | 3.796979603 | 2.584525017 |
| miR-196b-3p | 1.100986712 | 0.944455804 | 2.108738834 |
| miR-19d-5p | 1.146884909 | 0.324773312 | 0.461103294 |
| miR-19d-3p | 464.8274892 | 216.6194789 | 279.8495117 |
| miR-107-5p | 33.73992263 | 24.4982826 | 52.37306064 |
| miR-107-3p | 1573.445283 | 1913.643613 | 3290.970631 |
| miR-103-5p | 0.932190434 | 0 | 0 |
| miR-103-3p | 881.0838253 | 904.7626911 | 1652.02075 |
| miR-100-5p | 26636.01942 | 23735.91093 | 30134.20947 |
| miR-100-3p | 158.1394746 | 98.28636826 | 85.25759977 |
| miR-143-5p | 17.69763752 | 22.6981773 | 29.84894093 |
| miR-143-3p | 100821.4121 | 28264.63287 | 75279.34167 |
| miR-1-3p | 2.150409204 | 2.353083914 | 2.01769146 |
| miR-144-5p | 1099.038593 | 518.975104 | 543.3363711 |
| miR-144-3p | 5796.149138 | 3897.890529 | 2834.83499 |
| miR-7b-5p | 3.982995491 | 1.661393242 | 4.687397423 |
| miR-7b-3p | 0.33897834 | 0.808102002 | 1.738092906 |
| miR-7a-5p | 2.63772129 | 11.37630063 | 15.85953946 |
| miR-7a-3p | 0.351696172 | 1.552644466 | 1.163032894 |
| miR-9-5p | 11.10512797 | 8.801101802 | 7.481612946 |
| miR-9-3p | 3.101676386 | 5.739529091 | 3.129039236 |
| miR-22a-5p | 255.2978613 | 248.2252548 | 314.8679581 |
| miR-22a-3p | 37108.02716 | 33391.9118 | 48038.82938 |
| miR-21-5p | 71255.67446 | 29013.31158 | 24969.11668 |
| miR-21-3p | 436.5798713 | 254.5817115 | 430.755422 |
| miR-218b-5p | 12.90881414 | 13.02315485 | 16.30130709 |
| miR-218a-5p | 42.71127079 | 50.77122543 | 27.96565458 |
| miR-218a-3p | 23.03609927 | 0.052851793 | 28.8592536 |
| miR-222-5p | 1146.262036 | 723.2826789 | 647.6736151 |
| miR-222-3p | 1951.898505 | 1216.572279 | 1646.258417 |
| miR-137-3p | 9.906108845 | 0 | 18.6085263 |
| miR-139-5p | 69.48827963 | 69.93609432 | 86.23506516 |
| miR-139-3p | 0.33897834 | 0.052851793 | 0.113366711 |
| miR-138-5p | 33.35215163 | 30.33202145 | 32.73417018 |
| miR-489-5p | 0.997165379 | 0.717723524 | 1.798594486 |
| miR-489-3p | 2.807903351 | 4.574431388 | 4.028336479 |
| miR-34-5p | 50.39726814 | 66.50986335 | 60.07432693 |
| miR-34-3p | 1.401118663 | 0.332435866 | 0.287235003 |
| miR-722-5p | 1669.394895 | 1789.820693 | 2185.007456 |
| miR-722-3p | 20599.5693 | 20671.44083 | 21138.94177 |
| miR-132b-5p | 2.814955159 | 3.577123791 | 2.464111865 |
| miR-727-5p | 5.213932093 | 3.305988359 | 1.163032894 |
| miR-727-3p | 4.269023826 | 2.29718693 | 0.845842105 |
| miR-724-5p | 77.24593505 | 64.63676547 | 55.24124698 |
| miR-724-3p | 0.423722925 | 0.215238449 | 0.219096974 |
| miR-132a-5p | 39.20034378 | 71.41535674 | 41.50214557 |
| miR-132a-3p | 11.54087584 | 19.78523831 | 18.81943046 |
| miR-729-3p | 0.16948917 | 0 | 0 |
| miR-124a-3p | 1.94773967 | 1.02029526 | 0.211460526 |
| miR-124b-3p | 1.042370684 | 1.846767561 | 2.743710419 |
| miR-18b-5p | 14.47942197 | 12.77343491 | 28.23008116 |
| miR-18b-3p | 0.990113571 | 0.434308174 | 0.400601714 |
| miR-18c-5p | 123.9426487 | 135.5196738 | 237.1920706 |
| miR-18c-3p | 2.352385846 | 1.801578322 | 2.827121344 |
| miR-18a-5p | 47.71156998 | 35.68910976 | 69.3199258 |
| miR-18a-3p | 3.642631368 | 1.529656803 | 2.660299494 |
| miR-26a-5p | 110148.2413 | 102837.5548 | 136353.1653 |
| miR-26a-3p | 274.6298187 | 263.240362 | 128.32751 |
| miR-187-5p | 0.286721227 | 0.05668307 | 0.052865132 |
| miR-187-3p | 4.879926886 | 8.116287599 | 4.890041487 |
| miR-184-5p | 0.16948917 | 0.336267143 | 0 |
| miR-184-3p | 241.6485185 | 147.623039 | 82.21311783 |
| miR-182-5p | 31.54361523 | 31.24975747 | 68.58256215 |
| miR-183-5p | 4.704771692 | 3.365716619 | 9.049933968 |
| miR-183-3p | 0 | 0 | 0.052865132 |
| miR-200b-5p | 4.652514579 | 3.459926374 | 2.546342776 |
| miR-200b-3p | 354.2090184 | 226.0053923 | 313.8660152 |
| miR-200a-5p | 26.74981871 | 26.04169234 | 18.23617763 |
| miR-200a-3p | 2938.84543 | 2364.362633 | 3144.460515 |
| miR-101b-5p | 1175.302808 | 524.3887118 | 614.9410286 |
| miR-101b-3p | 16974.68791 | 14829.57618 | 17324.87051 |
| miR-16a-5p | 383.0010219 | 224.7074817 | 196.6900979 |
| miR-16a-3p | 0.547313898 | 0 | 0.332463686 |
| miR-16b-5p | 2724.200818 | 2405.476534 | 2438.331166 |
| miR-16b-3p | 54.14082639 | 52.43114566 | 35.65587894 |
| miR-16c-5p | 486.6993368 | 401.0531084 | 348.8369065 |
| miR-16c-3p | 19.91938059 | 23.63349753 | 28.10026154 |
| miR-96-5p | 0.944908266 | 0.717723524 | 1.510769476 |
| miR-93-5p | 179.7945491 | 142.1393131 | 169.7417601 |
| miR-93-3p | 7.155312846 | 3.247733116 | 7.475156512 |
| miR-99-5p | 5094.511588 | 3914.54693 | 5109.34087 |
| miR-99-3p | 22.24633077 | 19.50260904 | 23.54855017 |
| miR-460-5p | 31.12902279 | 27.91685101 | 37.49619571 |
| miR-460-3p | 313.3235279 | 260.0021611 | 174.9800272 |
| miR-30d-5p | 9044.931139 | 10826.769 | 15302.06071 |
| miR-30d-3p | 0.925138626 | 0.6533779 | 0.461103294 |
| miR-30b-5p | 1236.151857 | 739.8249711 | 1400.9988 |
| miR-30b-3p | 2.00704859 | 2.114857802 | 0.876387898 |
| miR-27a-5p | 36.44111019 | 22.68069224 | 32.1109651 |
| miR-27a-3p | 687.3392209 | 680.0142409 | 702.6440282 |
| miR-133a-5p | 2.195614508 | 0.955949636 | 1.103121321 |
| miR-133a-3p | 49.98292149 | 39.45495053 | 61.34843379 |
| miR-23a-5p | 1.563556025 | 0.11336614 | 0.876977905 |
| miR-23a-3p | 634.8102961 | 406.0740358 | 420.7242808 |
| miR-23b-5p | 0.293080143 | 0.381456382 | 0.173868291 |
| miR-23b-3p | 296.163258 | 270.7530855 | 378.3598739 |
| miR-193a-5p | 2.469617903 | 1.273060393 | 0.67256382 |
| miR-193a-3p | 488.2047239 | 372.7667906 | 287.2911387 |
| ccr-let-7g-5p | 2310.737665 | 3476.248307 | 2031.030604 |
| ccr-let-7g-3p | 133.434288 | 95.84221894 | 129.72498 |
| miR-126-5p | 15572.68816 | 19244.68809 | 20235.65567 |
| miR-126-3p | 12949.18644 | 12416.29971 | 20758.24405 |
| ccr-let-7b-5p | 1847.855659 | 1638.759136 | 1179.075465 |
| ccr-let-7b-3p | 177.6579193 | 137.6867957 | 94.11488615 |
| miR-122-5p | 17339.36285 | 17176.50449 | 15943.39502 |
| miR-122-3p | 1287.458573 | 1020.399369 | 1283.637214 |
| ccr-let-7a-5p | 22235.4375 | 29026.94196 | 16086.75854 |
| ccr-let-7a-3p | 363.997001 | 180.6654233 | 171.5702431 |
| ccr-let-7j-5p | 1150.86445 | 1053.982551 | 829.1673309 |
| ccr-let-7j-3p | 220.021327 | 203.6190214 | 294.8433507 |
| miR-129-5p | 1.603095306 | 2.2673228 | 7.970902006 |
| miR-129-3p | 0.89901007 | 0.62351377 | 4.607492899 |
| ccr-let-7i-5p | 1516.459395 | 760.7041136 | 520.3440861 |
| ccr-let-7i-3p | 36.23208174 | 15.58155556 | 27.97339195 |
| miR-499-5p | 4.221739847 | 3.781654495 | 3.823332388 |
| miR-499-3p | 1.576273858 | 0.785900426 | 1.163622901 |
| miR-128-5p | 14.46670414 | 20.11914719 | 10.52606124 |
| miR-128-3p | 2092.6487 | 3849.531846 | 3354.91237 |
| miR-301a-5p | 11.915929 | 8.999542124 | 6.288034259 |
| miR-301a-3p | 1297.730335 | 701.132827 | 764.3163292 |
| miR-454a-5p | 0.684315595 | 0.219069726 | 0.340100134 |
| miR-454a-3p | 143.6565882 | 82.29565214 | 88.57050377 |
| miR-454b-5p | 0.912420794 | 0.721554802 | 0.695473165 |
| miR-454b-3p | 985.5566441 | 710.3535068 | 533.7061861 |
| miR-221-5p | 105.7445341 | 40.31108865 | 65.03198372 |
| miR-221-3p | 632.8745813 | 501.3425548 | 871.3540759 |
| miR-375-5p | 10.84742976 | 20.03495825 | 39.31527223 |
| miR-375-3p | 583.7026727 | 1450.338288 | 2228.926948 |
| miR-210-5p | 273.5008706 | 250.7117465 | 310.2725681 |
| miR-210-3p | 438.9332297 | 302.5974841 | 516.0009344 |
| miR-217-5p | 8696.841837 | 6725.200088 | 2519.511443 |
| miR-217-3p | 37.49909316 | 28.71041399 | 16.45049602 |
| miR-27d-5p | 148.5295862 | 125.5911002 | 121.310574 |
| miR-27d-3p | 133.5523358 | 242.8356538 | 277.8753462 |
| miR-27c-5p | 40.44538399 | 14.17901783 | 13.96701485 |
| miR-27c-3p | 3099.805937 | 2792.383627 | 2486.906464 |
| miR-7133-5p | 1.433606136 | 1.431615772 | 2.42651963 |
| miR-7133-3p | 98.98738566 | 87.24132794 | 340.725513 |
| miR-7132-5p | 16.68775431 | 5.200204262 | 9.499304407 |
| miR-7132-3p | 0.827676209 | 0.109534863 | 0.370055921 |
| miR-457a-5p | 1260.48465 | 810.6385413 | 615.4036328 |
| miR-457a-3p | 19.76260925 | 2.991922792 | 3.476775819 |
| miR-457b-5p | 73.40711596 | 48.93310482 | 100.9233789 |
| miR-457b-3p | 19.85937878 | 0.487159967 | 11.6756249 |
| miR-155-5p | 69.80540966 | 22.72725534 | 45.15278968 |
| miR-17-5p | 792.2276703 | 697.347653 | 847.8290251 |
| miR-17-3p | 2.371462594 | 4.58897041 | 4.677990954 |
| miR-146a-5p | 52756.40454 | 12962.54046 | 55778.43509 |
| miR-146a-3p | 117.8631327 | 151.0762801 | 277.9753109 |
| miR-130a-5p | 21.90804532 | 17.35552885 | 16.13448524 |
| miR-130a-3p | 681.8584261 | 566.56382 | 557.3063205 |
| miR-130b-5p | 1675.974396 | 1826.696401 | 1434.186843 |
| miR-130c-5p | 20.78867475 | 24.36428706 | 22.26100771 |
| miR-130c-3p | 3736.61167 | 4294.075812 | 3537.367054 |
| miR-15a-5p | 849.6870246 | 638.8407255 | 928.8984457 |
| miR-15a-3p | 16.60867575 | 16.32226674 | 27.9034839 |
| miR-15b-5p | 4675.591648 | 2345.511108 | 1783.748959 |
| miR-15b-3p | 60.71658365 | 32.11522947 | 27.2667423 |

**Table S2** Primer and annealing temperature for qRT-PCR validation

| Name | Length | Primer sequence (5'to3') | annealing temperature |
| --- | --- | --- | --- |
| miR-125b-5p | 22 | tccctgagaccctaacttgtga | 55.5°C |
| miR-137-3p | 21 | ttattgcttaagaatacgcgt | 56.6°C |
| miR-217-5p | 22 | tactgcatcaggaactgattgg | 56°C |
| miR-365-3p | 22 | taatgcccctaaaaatccttat | 57°C |
| miR-551-3p | 21 | gcgacccatccttggtttctg | 57°C |
| miR-7133-3p | 21 | tagtttgattcacagcacaag | 58.5°C |
| miR-143-3p | 22 | tgagatgaagcactgtagctcg | 54°C |
| miR-146a-5p | 23 | tgagaactgaattccatagatgg | 55.9°C |
| miR-20a-3p | 23 | actgcagtgtgagcacttgaagt | 55.5°C |
| miR-21-5p | 23 | tagcttatcagactggtgttggc | 55.5°C |
| miR-457b-3p | 22 | tccagtattgctgttctgctgt | 55.5°C |
| U6 | 24 | atggactatcatatgcttaccgta | 65°C |
